# Identification of programmed cell death and mitochondria correlated biomarkers for Kawasaki disease by integrated bioinformatics and machine-learning algorithms with RT-qPCR verification

**DOI:** 10.3389/fgene.2026.1677146

**Published:** 2026-02-11

**Authors:** Tengyang Wang, Chengyi Wang, Yiwei Chen, Xiaofeng Guo

**Affiliations:** 1 Department of Cardiology, Fujian Children’s Hospital (Fujian Branch of Shanghai Children’s Medical Center), College of Clinical Medicine for Obstetrics & Gynecology and Pediatrics, Fujian Medical University, Fuzhou, Fujian, China; 2 Department of Hematology, Fujian Children’s Hospital (Fujian Branch of Shanghai Children’s Medical Center), College of Clinical Medicine for Obstetrics & Gynecology and Pediatrics, Fujian Medical University, Fuzhou, Fujian, China

**Keywords:** drug prediction, enrichment analysis, Kawasaki disease, mitochondrial, pediatrics, programmed cell death, vasculitis

## Abstract

**Background:**

The interplay between programmed cell death and mitochondrial dysfunction is a central mechanism in Kawasaki disease pathogenesis, yet the specific molecular connectors within this network are poorly defined. This study aimed to identify and validate integrated biomarkers that bridge these processes, offering new diagnostic and mechanistic insights.

**Methods:**

Using the GSE73461 dataset as a training set, we identified Kawasaki disease-related differentially expressed genes. These were intersected with curated programmed cell death-related and mitochondrial-related gene sets to obtain candidates. Subtype-specific analysis was performed to clarify which programmed cell death processes were most closely linked to mitochondrial dysfunction. A protein-protein interaction network was constructed, and hub genes were screened using multiple algorithms. Biomarker selection was refined via two machine learning models. Diagnostic performance was evaluated using the independent GSE68004 validation set, receiver operating characteristic analysis, and a composite nomogram. Immune infiltration, functional enrichment, regulatory network construction, drug prediction, and molecular docking were also conducted. Finally, biomarker expression was validated in clinical blood samples using reverse transcription quantitative polymerase chain reaction.

**Results:**

We identified 63 candidate genes. Five biomarkers—CD177, Matrix Metallopeptidase 9, Nuclear Factor Erythroid 2, Colony Stimulating Factor 3 Receptor, and Suppressor Of Cytokine Signaling 3—were consistently upregulated in Kawasaki disease and showed high diagnostic accuracy (area under the curve >0.94 in both datasets). These biomarkers are functionally anchored in a network where specific programmed cell death subtypes, strongly correlated with mitochondrial genes, converge on inflammatory pathways. A diagnostic nomogram integrating all five biomarkers achieved an area under the curve of 0.986. Clinical validation confirmed significant upregulation of CD177, Matrix Metallopeptidase 9, and Suppressor Of Cytokine Signaling 3, while Nuclear Factor Erythroid 2 and Colony Stimulating Factor 3 Receptor exhibited no significant differential expression. All five biomarkers were enriched in the Fc gamma receptor–mediated phagocytosis pathway and correlated cohesively with key immune cells. Decitabine and Ibuprofen were highlighted as potential therapeutic candidates.

**Conclusion:**

This study defines a novel five-biomarker panel that functionally links mitochondrial dysfunction, dysregulated programmed cell death, and immune activation in Kawasaki disease, providing an integrated, mechanism-based framework for improving diagnosis and guiding future targeted therapies.

## 1 Introduction

Kawasaki disease (KD), sometimes referred to as mucocutaneous lymph node syndrome, is a self-limiting systemic vasculitis that mostly affects children under 5 years of age. The incidence of KD ranges from 3.4 to 218.6 cases per 100,000 children (McCrindle et al., 2017). Key clinical characteristics of KD include nonspecific fever, bilateral bulbar conjunctival injection, alterations of the oral mucosa, rash, changes in the extremities, and cervical lymphadenopathy (Kawasaki et al., 1974). Approximately 20%–25% of untreated KD patients develop coronary artery abnormalities, the most significant complications linked to the disease. These abnormalities can manifest as dilation and aneurysms of the coronary arteries, which may result in serious consequences such as luminal thrombosis, coronary artery stenosis, occlusion, myocardial ischemia, myocardial infarction, or even death (McCrindle et al., 2017). Consequently, coronary artery complications make KD the most prevalent acquired heart condition in children within developed countries, significantly impacting their health and wellbeing (McCrindle et al., 2017). The pathophysiology of KD suggests a complex interplay between genetic predispositions and various environmental factors (McCrindle et al., 2017; Onouchi, 2018; Manlhiot et al., 2018).

Programmed cell death (PCD) is an active, regulated cellular death process crucial for maintaining homeostasis, encompassing distinct forms such as apoptosis, necroptosis, autophagy, ferroptosis, and pyroptosis (Qin et al., 2023). Dysregulation of PCD is implicated in various diseases. In KD, genes involved in autophagy may significantly influence disease progression (Zhu et al., 2023), and endothelial cell apoptosis contributes to coronary artery injury (Wen et al., 2023). Mitochondria, double-membraned organelles central to cellular energy metabolism and immune cell function, are increasingly recognized for their role in inflammation (Faas and de Vos, 2020). Mitochondrial damage-associated molecular patterns (mtDAMPs) can activate inflammasomes, exacerbating the inflammatory response in KD (Beckley et al., 2022). Furthermore, disruptions in mitochondrial dynamics (e.g., excessive fission, mitophagy) can trigger various PCD pathways (Cheng et al., 2021; Nguyen et al., 2023). Our previous research in a mouse model demonstrated that blocking PINK1/PARKIN-mediated mitophagy alleviated KD symptoms by reducing inflammatory cytokines and mitochondrial dysfunction, underscoring the critical interplay between mitochondria and PCD in KD pathogenesis (Wang et al., 2024). Emerging research utilizing diverse murine models (e.g., induced by CAWS, LCWE, or FK565) highlights that KD-like vasculitis can be triggered via different innate immune pathways, with mitochondrial reactive oxygen species (ROS) and subsequent NLRP3 inflammasome activation being a common effector mechanism (Hara and Sakai, 2025).

Recent studies have deepened our understanding of these mechanisms. For instance, mitochondrial DNA (mtDNA) released via the mitochondrial permeability transition pore (mPTP) has been shown to activate the cyclic GMP–AMP synthase–stimulator of interferon genes (cGAS-STING) pathway, significantly exacerbating inflammation in acute KD (Wei et al., 2024). Additionally, specific PCD pathways like pyroptosis, regulated by inflammasomes such as NLRP3 and AIM2, are directly implicated in driving vascular endothelial damage in KD (Hara and Sakai, 2025; Ohnishi et al., 2025). These findings highlight the intricate crosstalk between mitochondrial damage, specific PCD pathways, and the resulting inflammatory storm in driving KD pathology.

While a link between PCD and mitochondrial function in KD is established, the specific molecular mediators connecting these processes and their utility as integrated biomarkers remain underexplored. Previous biomarker studies have often focused on single genes or pathways. To bridge these gaps, this study employed integrated bioinformatics and machine learning to systematically identify and validate biomarkers that are correlated with both PCD and mitochondrial function in KD. We hypothesized that such biomarkers could serve as key nodes in the pathogenic network, offering novel diagnostic and therapeutic insights. Our analysis encompassed differential expression screening, functional enrichment, immune infiltration profiling, drug prediction, and validation using independent datasets and clinical samples. This approach aims to provide a more comprehensive, mechanism-linked understanding of KD, moving beyond isolated biomarker discovery towards elucidating their roles within the interconnected landscape of PCD, mitochondrial dysfunction, and immune dysregulation.

## 2 Materials and methods

### 2.1 Data collection

Datasets GSE73461 and GSE68004 were retrieved from the Gene Expression Omnibus (GEO, https://www.ncbi.nlm.nih.gov/geo/) database using the GPL10558 platform. This study employed a training-validation strategy. The GSE73461 dataset, comprising whole blood samples from 78 KD patients and 55 control individuals, served as the training set for initial biomarker discovery. The GSE68004 dataset, with whole blood samples from 76 KD patients and 37 controls, was used as an independent validation set. A total of 1,548 PCD-related genes (PCD-RGs) and 1,136 mitochondrial-related genes (mito-RGs) were compiled from the literature (Qin et al., 2023).

### 2.2 Differential expression and functional enrichment analysis

Differentially Expressed Genes in KD (KD-DEGs) between KD patients and controls in the GSE73461 dataset were determined by making use of the Limma package (v 3.52.4) (Ritchie et al., 2015) for differential expression analysis, applying criteria of |log2FoldChange(FC)| ≥ 0.5 and p.adj <0.05. Following this, the PCD and mitochondrial scores for the samples in GSE73461 were computed by means of the single sample gene set enrichment analysis (ssGSEA) method with PCD-RGs and mito-RGs as background gene sets. Additionally, Pearson correlation analysis was carried out for the KD-DEGs and the two scores, applying a screening threshold of |R| ≥ 0.3 and p < 0.05. The candidate genes were considered as the intersection of the screened PCD-RGs and mito-RGs. Gene Ontology (GO) and Kyoto Encyclopedia of Genes and Genomes (KEGG) enrichment analyses of these candidate genes were done using the KOBAS-i database (https://kobas.cbi.pku.edu.cnn/) to understand their associated biological functions and pathways, with a significance level of p < 0.05.

### 2.3 Subtype-specific analysis of programmed cell death and mitochondrial correlation

To further analyze the relationship between specific PCD subtypes and mitochondrial function in KD, we conducted subtype-specific enrichment and correlation analyses. In addition, the enrichment scores of 18 PCD subtypes (Ye et al., 2025) and mitochondrial gene sets were calculated by the ssGSEA method using the GSVA software package (v 1.50.0) (Hänzelmann et al., 2013). The parameters are set as follows: method = “ssgsea,” kcdf = “none,” ssgsea.norm = TRUE. Pearson correlation analysis was conducted between the ssGSEA scores of each PCD subtype and mitochondria. PCD subtypes with strong mitochondrial correlation were screened out with a threshold of p < 0.05 and cor >0.3. Further calculate the intersection between the gene sets of these related subtypes and KD-DEGs. KEGG enrichment analysis was performed on the obtained crossover gene set with a threshold of p < 0.05 to verify whether the core subtype genes were significantly enriched in mitochondrial-related pathways and KD-related inflammatory pathways.

### 2.4 Protein-protein interaction (PPI) network and machine learning

By utilizing the STRING database (https://cn.string-db.org/), the PPI network for the candidate genes was created. Subsequently, the cytoHubba plugin in Cytoscape (v 3.8.2) (Shannon et al., 2003) was employed to identify the top 20 genes using six algorithms Maximal Clique Centrality, Maximum Neighborhood Component, Degree, Edge Percolated Component, Closeness, and Radiality. The junction of genes found by these six algorithms was designated as hub genes for later machine learning analyses. There were two machine learning models that we utilized, which were the Least Absolute Shrinkage and Selection Operator (LASSO) and Support Vector Machine with Recursive Feature Elimination (SVM-RFE). The overlapping feature genes derived from these two models were considered as candidate biomarkers. Specifically, LASSO regression analysis was done on the hub genes by means of the glmnet package (v 4.1-4) (Friedman et al., 2010), selecting genes that were not penalized to zero while attaining little error. SVM-RFE analysis regarding the hub genes was performed with the e1071 package (v 1.7-16), where genes were recursively eliminated. Ultimately, feature genes from the SVM-RFE model that produced the minimum error rate were selected.

### 2.5 Expression level verification and receiver operating characteristic (ROC) analysis

The expression levels of candidate biomarkers in the GSE73461 (training) and GSE68004 (validation) datasets were validated, and their diagnostic accuracy was assessed by means of ROC curves. Biomarkers that demonstrated consistent expression patterns and significant differences between the KD and control groups in both datasets were determined to be valid biomarkers (p < 0.05) value >0.7 was also considered as the only biomarker with area under the curve (AUC) in the ROC analysis, which was only considered as reliable biomarkers.

### 2.6 Construction and assessment of a nomogram

The rms package (v 6.7-1) was used for the creation of the nomogram on the basis of the identified biomarkers. To assess the accuracy of the nomogram, ROC analysis, calibration curves, and decision curve analysis (DCA) were conducted.

### 2.7 Immune infiltration analysis

The scores of 28 immune cells in the training set samples were calculated using ssGSEA, with the immune-related genes serving as the background gene set (Charoentong et al., 2017). Subsequently, a comparison of scores was made between the KD and the control groups to identify any immune cells that showed differences. Also, the correlation of immune cells and biomarkers was computed.

### 2.8 The relevance of gene set enrichment analysis (GSEA) to inflammatory responses

In the GSE73461 dataset, KD patients were separated into high and low expression groups depending on the median expression levels of each biomarker. GSEA was run through the use of the GSEA software (v 4.2.3) (Subramanian et al., 2005), focusing on GO and KEGG background gene sets with a false discovery rate (FDR) of less than 0.05. The hallmark inflammatory response gene set was gathered from the Molecular Signatures Database (MSigDB, https://www.gsea-msigdb.org/gsea/msigdb). For the training dataset GSE73461, the Gene Set Variation Analysis (GSVA) package (v 1.50.0) (Hänzelmann et al., 2013) was applied to compute enrichment scores for all samples based on the inflammatory response gene set. Subsequently, Pearson correlations were computed between the biomarkers and the scores related to inflammatory genes. A p-value less than 0.05 was established as the criterion for identifying statistical significance.

### 2.9 Construction of networks

Biomarkers were input into the DisGeNET database (http://www.disgenet.org), applying a screening criterion of a score ≥0.1 to identify diseases associated with these biomarkers. Transcription factors (TFs) linked to the biomarkers were obtained from the hTFtarget database (http://bioinfo.life.hust.edu.cn/hTFtarget).Using criteria set at 0.6 and p < 0.05, the Pearson correlation coefficient was computed in order to examine the interactions between TFs and biomarkers. After that, the outcomes were inputted into Cytoscape (v 3.8.2) (Shannon et al., 2003) for the purpose of visualizing the TF-mRNA and disease-mRNA networks.

### 2.10 The prediction of drugs and molecular docking

The Enrichr package (v 3.2) (Kuleshov et al., 2016) was deployed to perform enrichment analysis on the biomarker gene set within the Drug Signatures Database (DSigDB, http://tanlab.ucdenver.edu/DSigDB) to identify candidate drugs that target these biomarkers. The 3D structure of the target drug was acquired as a ligand from PubChem (https://pubchem.ncbi.nlm.nih.gov/) and underwent energy minimization using ChemBioOffice software. The crystal structure of the receptor protein associated with the biomarker was retrieved from the Protein Data Bank (PDB) (https://www.rcsb.org/), and molecular docking between the target drug and the receptor protein was conducted using AutoDockTools (v 1.5.7) (Morris et al., 2009).

### 2.11 Expression validation of biomarkers

In order to validate our results further, we used reverse transcription quantitative polymerase chain reaction (RT-qPCR) to compare the amounts of biomarkers expressed in clinical samples. Blood samples were taken from five control volunteers and five KD patients at Fujian Children’s Hospital. Informed consent was acquired from all participants through signed consent forms, and the study obtained ethical approval from the Ethics Committee of Fujian Children’s Hospital (No. 2024ETKLRK08006). According to manufacturer instructions, RNA extraction and purification was carried out from the samples using TRIzol (Ambion, Austin, United States). Then, using the SureScript First-Strand cDNA Synthesis Kit (Servicebio, Wuhan, China), the extracted RNA was reverse transcribed into complementary DNA (cDNA). The resulting cDNA was quantified with the 2x Universal Blue SYBR Green qPCR Master Mix (Servicebio) using specific primer sequences (refer to Supplementary Table S1). Under the following thermal cycling conditions, RT-qPCR amplification was carried out: Firstly, an initial denaturation step was carried out at 95 °C for a duration of 1 min. Then, 40 cycles were performed, with each cycle consisting of denaturation at 95 °C for 20 s, annealing at 55 °C for 20 s, and extension at 72 °C for 30 s. For the data analysis, the 2^−ΔΔCT^ method was employed, in which GAPDH was used as the internal reference gene.

### 2.12 Statistical analysis

The R software (v 4.2.2) was chosen for the purpose of data processing and analysis. For the assessment of statistical significance, we defined the threshold as p < 0.05.

## 3 Results

### 3.1 Acquisition of 63 candidate genes

**FIGURE 1:**
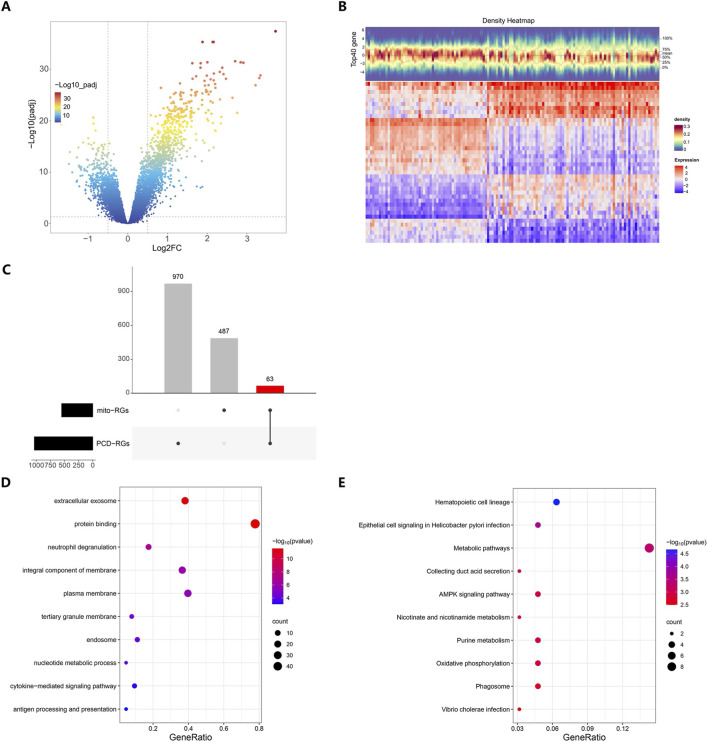
Acquisition of candidate genes. **(A)** Volcano plot for differential gene analysis of the KD and Normal groups in dataset GSE73461. **(B)** Heatmap of top 20 differentially expressed genes between the KD and Normal groups in dataset GSE73461. **(C)** Upset plot of mito-RGs and PCD-RGs. **(D)** Bubble chart of GO enrichment results. **(E)** Bubble chart of KEGG enrichment results.

There were 1,570 KD-DEGs found in the GSE73461 dataset. Among them, 1,048 genes showed an upregulation trend, while 522 genes experienced downregulation (Figures 1A,B). The correlation analysis selected a total of 1,033 PCD-RGs and 550 mito-RGs associated with KD, respectively. Their intersection yielded 63 candidate genes related to extracellular exosome, hematopoietic cell lineage, and metabolic pathways, etc. (Figures 1C–E).

### 3.2 Identification of PCD subtypes strongly associated with mitochondria and their enriched pathways

**FIGURE 2:**
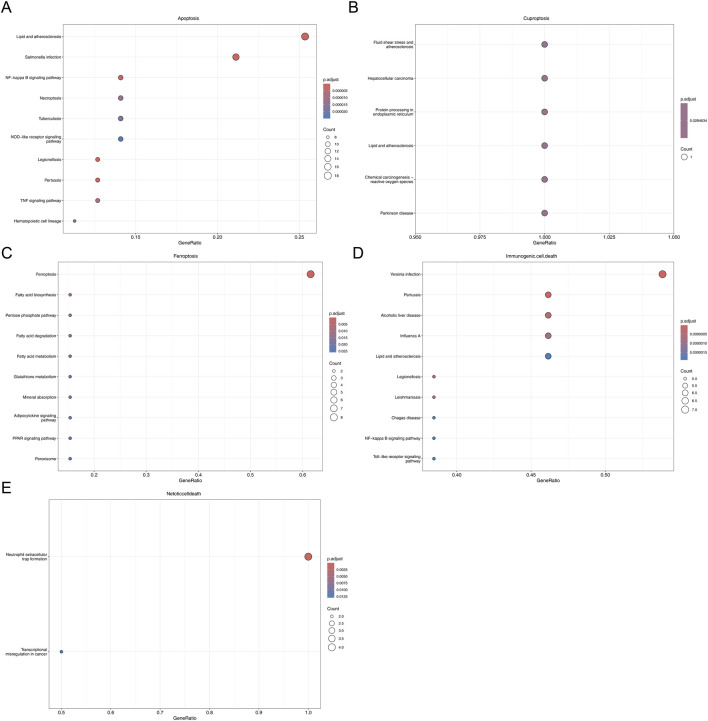
Subtype-specific analysis of programmed cell death and mitochondrial correlation. **(A)** Analysis of KEGG enrichment pathways in apoptosis (Top 10). The horizontal axis represents GeneRatio, which is the ratio of genes enriched in the entry to the total number of input genes. The size of the circles in the graph represents the number of genes enriched in the entry, and the color represents the significance of the entry, with blue to red indicating an increase in significance. **(B)** Analysis of KEGG enrichment pathways in cuproptosis. **(C)** Analysis of KEGG enrichment pathways in ferroptosis (Top 10). **(D)** Analysis of KEGG enrichment pathways in immunogenic cell death. **(E)** Analysis of KEGG enrichment pathways in netoticcelldeath.

Through correlation analysis of ssGSEA scores, six PCD subtypes were found to be closely associated with mitochondrial genes, namely Apoptosis, Ferroptosis, immunogenic cell death, netotic cell death, cuprotosis, and Oxeiptosis. The intersection of the gene set of each subtype with KD-DEGs yielded different subsets of candidate genes (Supplementary Table S2). KEGG enrichment analysis showed that these gene subsets were significantly enriched in pathways related to mitochondrial processes and KD-associated inflammation. Specifically, the core gene set of Apoptosis was enriched in 57 pathways, among which the pathways related to both mitochondria and KD-associated inflammation included the TNF signaling pathway, Necroptosis, Apoptosis, MAPK signaling pathway, and Cytosolic DNA-sensing pathway (Figure 2A); the core gene set of Cuprotosis was enriched in 6 pathways, with the common related pathways being Protein processing in endoplasmic reticulum and Chemical carcinogenesis - reactive oxygen species (Figure 2B); the core gene set of Ferroptosis was enriched in 6 pathways, and the common related pathways were Ferroptosis, Glutathione metabolism, and Peroxisome (Figure 2C); the core gene set of Immunogenic cell death was enriched in 56 pathways, with 10 common related pathways including the NF-κB signaling pathway, Toll-like receptor signaling pathway, and NOD-like receptor signaling pathway (Figure 2D); the core gene set of Netotic cell death was enriched in 2 pathways, and the only common related pathway was Neutrophil extracellular trap formation (NETosis) (Figure 2E).

### 3.3 Screening of candidate biomarkers

**FIGURE 3:**
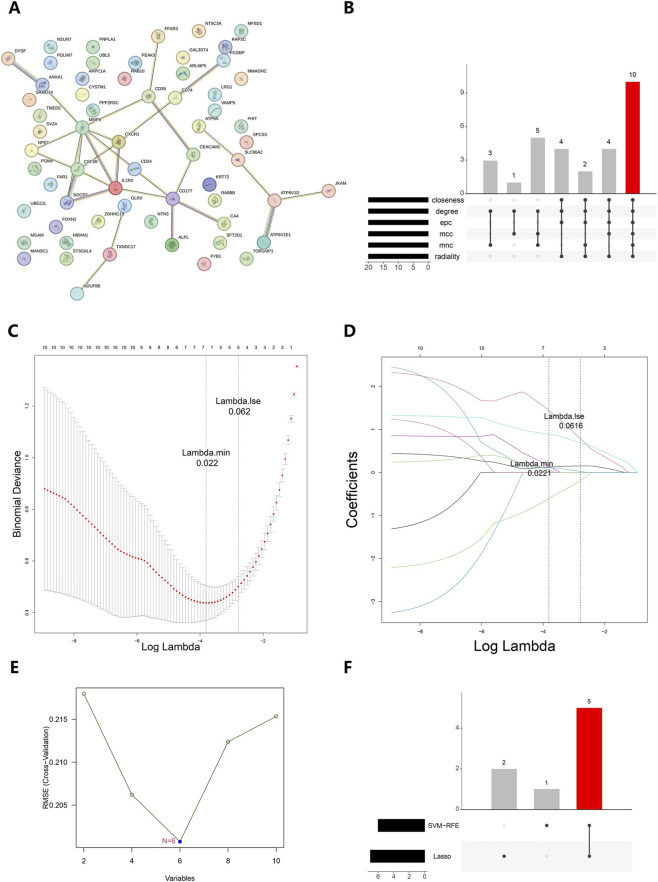
Screening of candidate biomarkers. **(A)** Analysis of PPI network of Intersecting genes. **(B)** Upset plot of cytoHubba. **(C)** Penalty parameter plot of the LASSO model. The horizontal axis represents the log lambda value, and the vertical axis represents the error of cross validation. The dashed line on the left is the position with the minimum error of cross validation. **(D)** Coefficient profiles of candidate genes in the LASSO model. The horizontal axis of the graph is log lambda, and the vertical axis is the coefficient of the gene. The left dashed line represents the gene and its coefficient corresponding to the optimal log lambda value. **(E)** Correlation diagram between RMSE and feature numbers in the SVM-RFE model. **(F)** Upset plot of the LASSO model and the SVM-RFE model. RMSE, root mean square error.

The PPI network included 63 candidate genes for protein interactions, such as interactions of MMP9, CSF3R, and SOCS3 (Figure 3A). Thereafter, 10 hub genes were screened via the intersection of the top 20 genes identified by six algorithms in the cytoHubba plug-in (Figure 3B). Next, these 10 hub genes were fed into the LASSO and SVM-RFE models. The LASSO model with minimum error identified seven feature genes (lambda-min = 0.022), which were NFE2, CD74, CD177, SOCS3, MMP9, ANXA1 and CSF3R (Figures 3C,D). The SVM-RFE model with the least error included six feature genes, namely CD177, MMP9, NFE2, CSF3R, SOCS3, and IL1R2 (Figure 3E). Consequently, Consequently, five genes—CD177, matrix metallopeptidase 9 (MMP9), nuclear factor, erythroid 2 (NFE2), colony stimulating factor 3 receptor (CSF3R), and suppressor of cytokine signaling 3 (SOCS3)—were identified as overlapping candidate biomarkers from the two models (Figure 3F).

### 3.4 CD177, MMP9, NFE2, CSF3R, and SOCS3 were reliable biomarkers for KD

**FIGURE 4:**
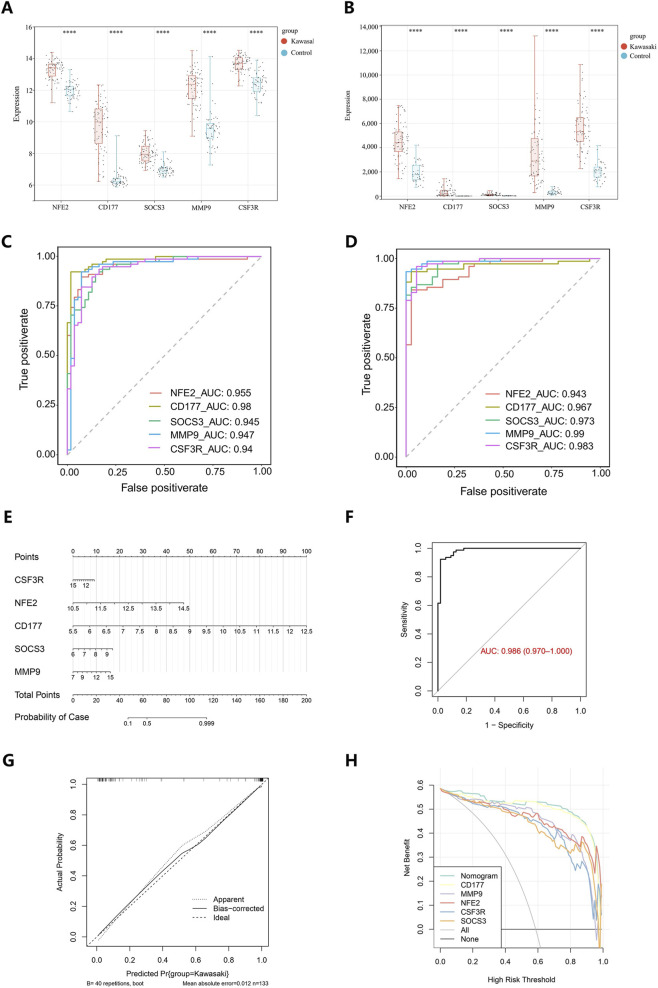
Diagnostic value assessment of biomarkers. **(A)** Expression of biomarkers in KD group and control group in the training set (GSE73461). **(B)** Expression of biomarkers in KD group and control group in the validation set (GSE68004). **(C)** ROC analysis of biomarkers in the training set (GSE73461). Different genes are indicated by different colors. **(D)** ROC analysis of biomarkers in the validation set (GSE68004). Different genes are indicated by different colors. **(E)** Nomogram established based on biomarkers. **(F)** ROC analysis of the nomogram. **(G)** Calibration curve analysis of thenomogram. **(H)** Decision curve analysis of the nomogram and biomarkers. KD, Kawasaki disease; AUC, area under curve; ROC, receiver operating characteristic curve.

CD177, MMP9, NFE2, CSF3R, and SOCS3 were significantly upregulated in KD samples in both GSE73461 and GSE68004 datasets (Figures 4A,B). The AUC values of CD177, MMP9, NFE2, CSF3R, and SOCS3 were 0.98, 0.947, 0.955, 0.94, and 0.945 in GSE73461 and were 0.967, 0.99, 0.943, 0.983, and 0.973 in GSE68004 respectively, demonstrating their reliability as biomarkers (Figures 4C,D).

A nomogram was established based on these biomarkers, and the total points were computed to predict the probability of KD (Figure 4E). The nomogram had an AUC of 0.986, which was very good predictive ability (Figure 4F). Additionally, the calibration curve demonstrated a slope close to 1, further confirming its accuracy (Figure 4G). Moreover, the net benefits derived from the nomogram surpassed those obtained from individual genes alone, underscoring its superior prediction efficacy (Figure 4H).

### 3.5 The relevance trends of these immune cells with biomarkers were consistent

**FIGURE 5:**
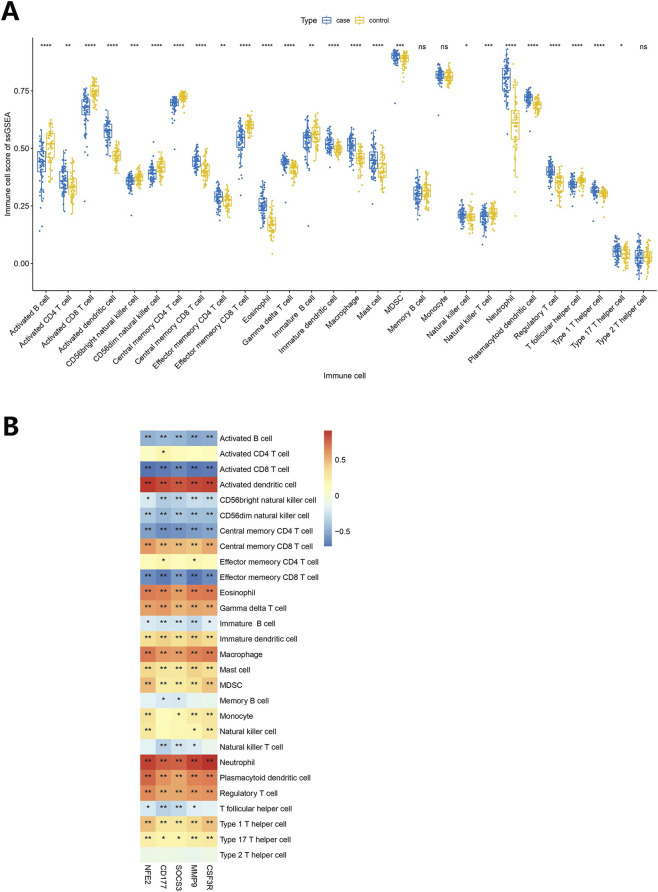
Analysis of immune landscape associated with KD. **(A)** Violin plot showing the distribution of 28 types of immune cells in acute KD and convalescent KD. **(B)** The relationship between five biomarkers and immune cell infiltration. The Wilcoxon test was applied to compare the proportions of immune cells between acute phase and convalescent phase in KD. The Spearman correlation test was employed to analyze the correlation of hub genes with 28 immune cells. KD, Kawasaki disease; *p < 0.05, **p < 0.01, ****p < 0.001.

The scores of 25 immune cells differed significantly between KD and control groups, including activated dendritic cells, activated CD8 T cells and others (Figure 5A). Through correlation analysis, it was found that the five biomarkers exhibited similar association characteristics in their correlation with specific immune cell subsets. Specifically, they were all significantly positively correlated with activated dendritic cells and neutrophils, while significantly negatively correlated with activated CD8 T cells (Figure 5B). This association pattern suggests that these biomarkers may jointly participate in regulating the KD immune microenvironment, including the activation of dendritic cells, the recruitment of neutrophils, and the functional inhibition of CD8 T cells.

### 3.6 These five biomarkers played a role in pathways associated with immunity and inflammation

**FIGURE 6:**
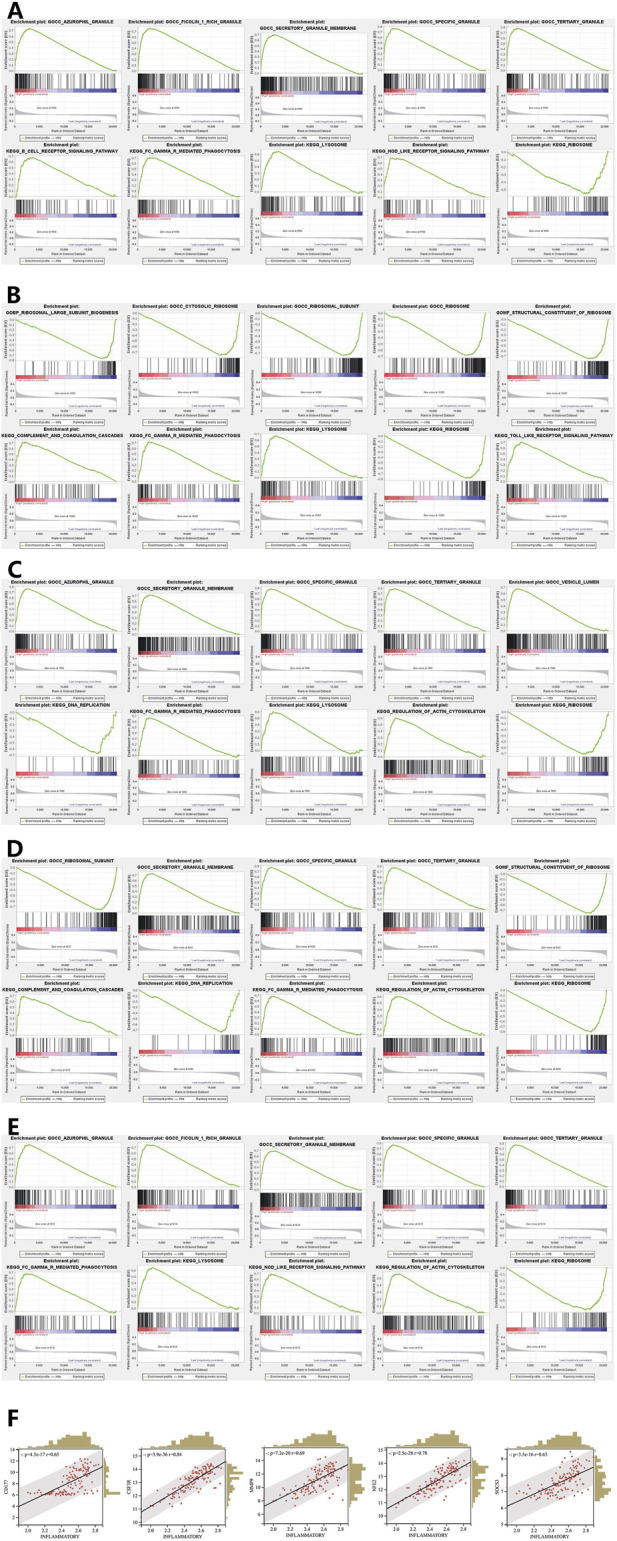
Gene set enrichment analysis and correlation between biomarkers and the inflammation-related gene score. **(A–E)** GSEA enrichment trend chart of CD177, CSF3R, MMP9, NFE2 and SOCS3. **(F)** Scatter plot of the correlation between biomarkers and inflammation-related gene scores.

CD177 showed significant enrichment in GO categories including tertiary granules, special granules, ficolin 1-rich granules, and others. In KEGG pathways, it showed significant enrichment in processes such as Fc gamma receptor (FcγR)-mediated phagocytosis, NOD-like receptor signaling pathways (Figure 6A). MMP9 was notably enriched in GO categories, including tertiary granule, special granule, and azurophil granule, as well as KEGG pathways such as DNA replication, FcγR-mediated phagocytosis, and regulation of the actin cytoskeleton (Figure 6B). Enrichment results for the remaining biomarkers were shown in Figures 6C–E. These five biomarkers were all involved in tertiary granule, special granule, FcγR-mediated phagocytosis, and ribosome functions or pathways related to Immunity and inflammation. The following analysis showed a notable positive correlation among all five biomarkers and the inflammation-related gene score (Figure 6F).

### 3.7 The complications of KD were associated with five biomarkers

**FIGURE 7:**
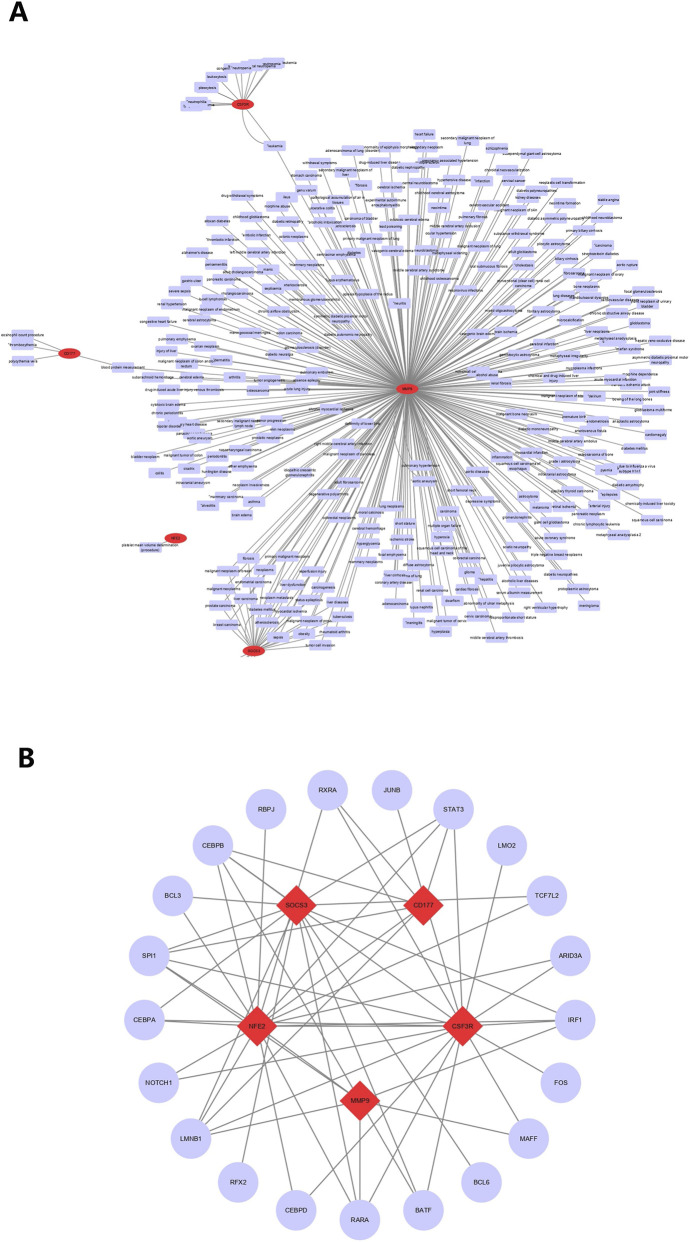
The interaction networks. **(A)** The interaction network of disease-mRNA (The red ovals represent hub genes, and the blue rectangles represent diseases that interact with biomarkers). **(B)** The interaction network of TFs-gene (The red diamonds represent biomarkers, and the blue circles represent TFs.). TFs, transcription factors; mRNAs, messenger RNA.

In disease-mRNA network, the biomarkers were associated with myocardial infarction, acute coronary syndrome, coronary arteriosclerosis and other diseases. Coronary artery disease and myocardial disease are the primary complications associated with KD (Figure 7A). There were 21 TFs predicted by five biomarkers, among which SP11 and LMNB1 were linked with all five biomarkers (Figure 7B).

### 3.8 Decitabine and Ibuprofen were effective target drugs for KD

**FIGURE 8:**
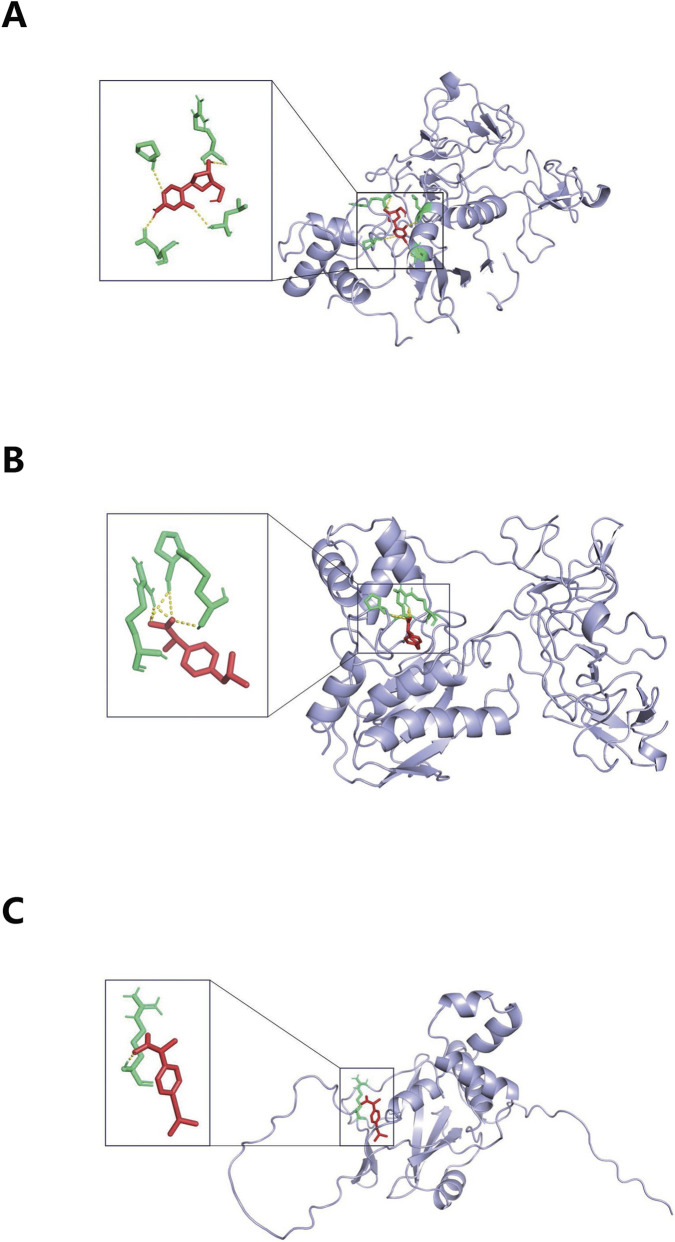
Visualization of molecular docking between the target drug and receptor protein of biomarkers. **(A)** Molecular docking between MMP9 and Decitabine. **(B)** Molecular docking between MMP9 and Ibuprofen. **(C)** Molecular docking between SOCS3 and Ibuprofen.

NICKEL SULFATE, Healon, Decitabine, Ibuprofen, Pyrrolidine dithiocarbamate, and Hydroquinone were target drugs for biomarkers (Table 1). The 3D structures of NICKEL SULFATE, Healon, and Pyrrolidine dithiocarbamate were not obtained in this study, and the remaining drugs were applied for molecular docking. The binding energies of MMP9 with Decitabine (−6.39 kcal/mol), MMP9 with Ibuprofen (−6.52 kcal/mol), and SOCS3 with Ibuprofen (−5.67 kcal/mol) were found to be below −5 kcal/mol, indicating a superior binding effect (Figures 8A–C).

**TABLE 1:** Target drugs for biomarkers.

Drug	P value	Odds ratio	Combined score	Genes
Nickel sulfate	2.28E-04	50.84	426.47	SOCS3; CSF3R; MMP9
Healon	3.24E-04	117.29	942.44	MMP9; CD177
Decitabine	5.08E-04	93.21	707.06	SOCS3; MMP9
Ibuprofen	5.43E-04	90.01	676.72	SOCS3; MMP9
Pyrrolidine dithiocarbamate	6.18E-04	84.24	622.4	NFE2; MMP9
Hydroquinone	7.23E-04	77.75	562.27	NFE2; MMP9

### 3.9 Expression of biomarkers in clinical samples

**FIGURE 9:**
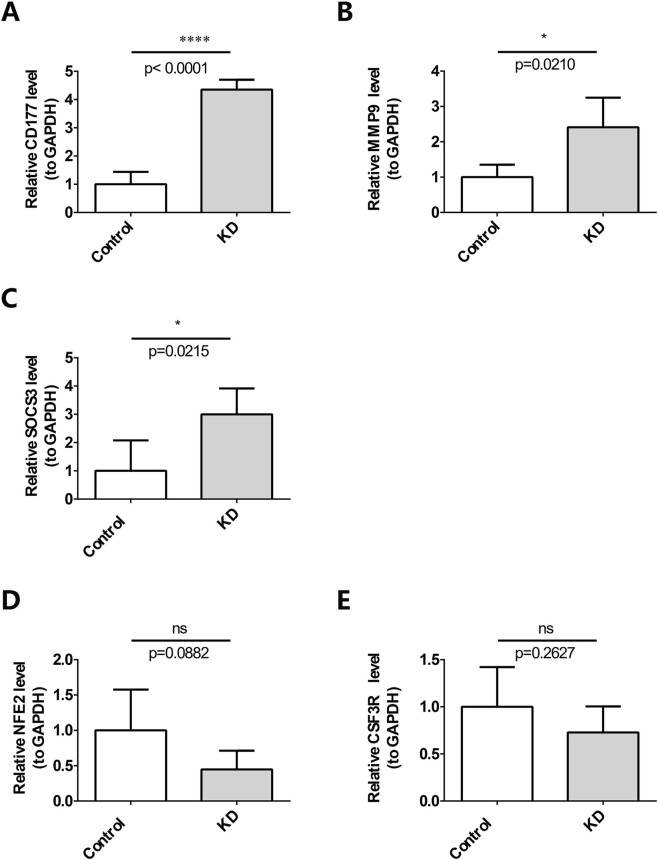
Validation of the differential expression of potential biomarkers, **(A)** CD177, **(B)** MMP9, **(C)** SOCS3, **(D)** NFE2, and **(E)** CSF3R, via RT-qPCR. *P < 0.05, ****P < 0.0001; ns, not significant.

The analysis results of the datasets revealed that the expression levels of these five biomarkers were found to be upregulated in the KD samples. The RT-qPCR results revealed significant overexpression of three out of the five biomarkers, namely CD177, MMP9, and SOCS3, in KD clinical samples (Figures 9A–C). However, NFE2 and CSF3R did not exhibit similar findings (Figures 9D,E).

## 4 Discussion

KD is the leading cause of acquired heart disease in children, yet its diagnosis and treatment are hampered by an incomplete understanding of its etiology and pathogenesis (McCrindle et al., 2017). Growing evidence implicates both PCD and mitochondrial dysfunction in KD development (Zhu et al., 2023; Beckley et al., 2022; Wang et al., 2024). However, the specific molecular connectors between these two fundamental processes in KD have been less clear. In this study, we integrated bioinformatics, machine learning, and clinical validation to identify five biomarkers—CD177, MMP9, NFE2, CSF3R, and SOCS3—that are significantly correlated with both PCD and mitochondrial function in KD. Our findings suggest these genes act as critical nodes in a pathogenic network linking mitochondrial stress, dysregulated PCD, FcγR pathway activation, and immune cell infiltration, ultimately contributing to vasculitis.

### 4.1 The PCD-mitochondria axis: a central pathogenic mechanism in KD

The interplay between PCD and mitochondrial dysfunction constitutes a pivotal axis in the pathogenesis of Kawasaki disease. Mitochondria, beyond their role in energy metabolism, are central regulators of both immune cell function and multiple PCD pathways. In the context of KD, several lines of evidence illustrate this crosstalk. First, mitochondrial damage and the subsequent release of damage-associated molecular patterns (mtDAMPs, such as mtDNA) act as potent inflammasome activators, fueling the systemic inflammatory response characteristic of acute KD (Beckley et al., 2022). A recent study demonstrated that mtDNA release via the mPTP activates the cGAS-STING pathway, significantly exacerbating inflammation in acute KD (Wei et al., 2024). This inflammatory milieu, rich in cytokines and oxidative stress, in turn creates a cellular environment primed for PCD. Second, specific PCD pathways are directly orchestrated by mitochondrial events. For instance, the integrity of the mitochondrial membrane is a key determinant in the intrinsic apoptosis pathway, while dysregulated mitochondrial fission and excessive mitophagy can drive other forms of PCD such as necroptosis and ferroptosis (Cheng et al., 2021; Nguyen et al., 2023). Notably, mitochondrial ROS are critical for activating the NLRP3 inflammasome, leading to pyroptosis, an inflammatory form of PCD strongly implicated in KD vasculitis models (Hara and Sakai, 2025). Our subtype-specific analysis reinforces this paradigm by demonstrating how distinct PCD pathways, converging on mitochondrial stress, are collectively channeled into the intense inflammatory response of KD. This analysis reveals that gene sets for key PCD subtypes—including apoptosis, immunogenic cell death, and NETosis—are significantly enriched in canonical inflammatory and stress-response pathways central to KD pathophysiology. This provides a mechanistic scaffold linking the broad concept of mitochondrial-PCD crosstalk to the specific, dysregulated signaling networks that drive vasculitis. Our previous experimental work also supports this link, demonstrating that in a KD mouse model, modulating PINK1/PARKIN-mediated mitophagy—a process at the nexus of mitochondrial quality control and cell fate—significantly influenced inflammatory outcomes and cellular damage (Wang et al., 2024). Recent proteomic evidence further underscores mitochondrial centrality, showing that in a KD mouse heart, a majority (67.57%) of proteins with altered lysine lactylation modifications are localized to mitochondria, indicating profound mitochondrial involvement in KD pathophysiology (Zhuo et al., 2025). Conversely, activation of PCD pathways, such as pyroptosis or apoptosis, can further exacerbate mitochondrial damage, creating a vicious cycle that perpetuates vascular endothelial injury, a hallmark of KD complications like coronary artery lesions. The biomarkers identified in our study likely operate within this interconnected network. Therefore, our findings of PCD- and mitochondria-correlated biomarkers do not merely represent parallel associations but likely pinpoint key molecular nodes within a feed-forward loop of mitochondrial dysfunction and dysregulated PCD that drives KD vasculitis.

### 4.2 The identified biomarkers as functional nodes in the network

Elaborating on this pathogenic axis, the five identified biomarkers can be interpreted as critical functional nodes or effectors within the interconnected network of KD pathology. They appear to mediate the vicious cycle connecting mitochondrial dysfunction, dysregulated PCD, and immune activation.

Although our clinical validation in a small Asian cohort did not show statistically significant overexpression for NFE2, its central identification and strong diagnostic performance in the European bioinformatics datasets suggest its potential mechanistic relevance. Within the proposed axis, NFE2 may act as a critical sensor and regulator of mitochondrial oxidative stress. Its bioinformatics-derived upregulation could represent a compensatory antioxidant response to severe mitochondrial stress and mtDNA release (Wei et al., 2024), given its encoded protein’s role as an oxidoreductase necessary for intracellular redox reactions and antioxidant defense (Hayes and Dinkova-Kostova, 2014). Failure of this response could exacerbate mitochondrial damage and promote PCD subtypes like ferroptosis, directly linking it to the core pathology. This role is supported by the central function of NFE2 in oxidative stress response and heme metabolism, supporting the inference that it may play an important, if indirect, regulatory function in vascular endothelial injury and immune dysregulation in KD (Yang et al., 2025), processes driven during the acute phase by ROS-induced mitochondrial dysfunction and subsequent cGAS-STING pathway activation (Wei et al., 2024).

MMP9, released by activated neutrophils, is a direct effector of vascular damage. Its significant upregulation and strong correlation with neutrophil infiltration position it downstream of mitochondrial-stress-induced immune activation and PCD, such as NETosis (Aymonnier et al., 2023). This aligns with findings that neutrophil extracellular traps, which contain MMP9, promote NLRP3 inflammasome activation and pyroptosis in KD (Jin et al., 2025), placing MMP9 at the intersection of innate immune activation, PCD, and tissue injury. Clinically, KD patients exhibit significantly elevated plasma levels of MMPs prior to immunoglobulin treatment (Wang et al., 2021), and such treatment has been found to inhibit MMP9 expression in monocytes via suppression of NF-κB and P38 MAPK activation (Zhou et al., 2015). This suggests MMP9’s crucial role in both the pathogenesis and the therapeutic mitigation of coronary artery lesions.

CD177 facilitates neutrophil transendothelial migration (Bai et al., 2017). It is reported to be highly expressed during the acute phase of KD (Abe and Matsuda, 2013; Huang et al., 2019), with higher levels associated with immunoglobulin resistance (Huang et al., 2019). By promoting the recruitment of neutrophils—cells whose effector functions (e.g., ROS production, NETosis) are mitochondria-dependent—CD177 amplifies the local cycle of mitochondrial damage and PCD at the vascular site, acting as a key recruiter within the pathogenic network.

SOCS3, elevated in KD, may reflect a feedback mechanism attempting to curb excessive inflammation triggered by mitochondrial DAMPs. Its interaction with the FcγR pathway, as highlighted by our GSEA, places it centrally in modulating immune complex-driven inflammation, a key component of the pathological loop. Notably, in the hyperinflammatory state of KD, the STAT3 pathway becomes aberrantly activated (Xu et al., 2025). This activation not only suppresses normal PCD processes such as apoptosis—leading to the accumulation of inflammatory cells and prolonged tissue damage—but may also further induce mitochondrial dysfunction (Wang et al., 2025). As a key negative regulator of STAT3 signaling, insufficient SOCS3 function would fail to terminate this signal. This failure could perpetuate inflammation by both hindering the normal PCD required to clear inflammatory cells and potentially exacerbating mitochondrial dysfunction (Chen et al., 2025; Li et al., 2025), thereby solidifying its role as a dysfunctional brake within the pathological loop.

Similarly, while the clinical validation result for CSF3R was not significant in our limited sample, its selection as a core biomarker warrants mechanistic consideration. CSF3R is a critical receptor regulating granulocyte production and function (Liongue et al., 2012; Tarlock et al., 2020), pointing to the source of the dysregulated immune infiltrate. Altered CSF3R signaling could prime neutrophils for enhanced mitochondrial dysfunction and PCD upon activation. Interestingly, in oncology, CSF3R signaling activates the JAK2/STAT3 pathway to promote disease progression (Feng et al., 2025). This parallel suggests a mechanism whereby CSF3R in KD might similarly influence immune cell behavior through pathways that intersect with mitochondrial metabolism and PCD regulation. Consequently, targeting the CSF3R-mitochondria axis may offer a novel therapeutic strategy.

This interconnected view is strongly supported by our immune infiltration analysis. The consistent correlation patterns shared by all five biomarkers with key immune cells (e.g., activated dendritic cells, neutrophils) underscore their cohesive role in shaping the KD immune microenvironment. This microenvironment, as our analysis suggests, is fundamentally fueled by the dysregulated interplay between mitochondrial and PCD dynamics, with the identified biomarkers serving as pivotal functional nodes within this network.

### 4.3 Clinical validation and therapeutic implications

RT-qPCR validation on clinical samples from an Asian population confirmed the significant overexpression of CD177, MMP9, and SOCS3 in KD patients. The lack of significant difference for NFE2 and CSF3R in this small cohort may be attributed to racial/ethnic heterogeneity (McCrindle et al., 2017), as evidenced by genetic variations in immune-related loci like FCGR across populations (Shrestha et al., 2011), and limited sample size, highlighting the need for larger, multi-ethnic validation studies. Specifically, the p-value for NFE2 expression difference was 0.0882, indicating a trend toward significance that may reach statistical significance with a larger sample size.

Drug prediction identified Decitabine and Ibuprofen as potential candidates. Ibuprofen, a common NSAID, requires caution in KD as it may competitively inhibit aspirin’s antiplatelet effect, potentially increasing the thrombotic risk in coronary aneurysms (Sohn and Kwon, 2008; Alvarez-Coca González et al., 2009). Decitabine, a DNA methyltransferase inhibitor used in hematological malignancies (Garcia-Manero et al., 2020), represents a novel potential immunomodulatory avenue for KD suggested by our analysis. More broadly, our network perspective suggests that therapeutic strategies aiming to break the cycle of mitochondrial dysfunction and specific PCD pathways—such as targeting mtDNA release, inflammasomes (Hara and Sakai, 2025), or ferroptosis—represent promising future avenues informed by our biomarker findings.

### 4.4 Innovation of the present study

This study provides several key advancements that move beyond isolated biomarker discovery to offer a mechanism-integrated perspective, underscoring its multidimensional innovative value.

First, while CD177 (Huang et al., 2019), MMP9 (Wang et al., 2021), and SOCS3 (Ohashi et al., 2019) have been individually associated with KD, this is the first report to systematically position them within an integrated framework linking dysregulated PCD and mitochondrial dysfunction in KD. For instance, we confirmed MMP9’s upregulation and high diagnostic value but further highlight its dual association with PCD processes like NETosis and mitochondrial-related inflammatory pathways, offering a more mechanistic context for its role in vascular injury. Similarly, we extend the understanding of SOCS3 and CD177 by linking their expression to immune cell infiltration patterns that are heavily dependent on mitochondrial metabolism and PCD fates.

Second, we identified NFE2 and CSF3R as novel potential KD-associated genes. NFE2, a key regulator of oxidative stress and implicated in PCD forms like ferroptosis and cuproptosis (Qin et al., 2023; Hayes and Dinkova-Kostova, 2014), and CSF3R, a critical regulator of granulocyte differentiation and function (Tarlock et al., 2020), have not been previously reported in the context of KD. Their significant upregulation and high AUC values in both European population datasets suggest they may be valuable biomarkers in specific populations, warranting further investigation.

Third, we constructed a robust multi-biomarker diagnostic nomogram (AUC = 0.986) that outperforms single biomarkers, offering a potentially more reliable tool for KD diagnosis.

Finally, our GSEA revealed that all five biomarkers converge on the FcγR-mediated phagocytosis pathway. While this pathway’s general link to KD is known (Shrestha et al., 2012; Nagelkerke et al., 2014), our study uniquely identifies a specific set of PCD/mitochondria-related genes as its key effectors. This inference aligns with genetic studies linking FCGR polymorphisms to KD susceptibility (Uittenbogaard et al., 2024; Shrestha et al., 2011). However, recent analyses suggest these genetic variants may not directly predict IVIG resistance or aneurysm risk (Uittenbogaard et al., 2024). This distinction underscores that our identified biomarkers likely operate as functional mediators within the FcγR pathway activation cascade, rather than merely reflecting genetic predisposition. Yan et al. demonstrated that SOCS3 is essential for enhancing the FcγR-mediated inflammatory response in macrophages (Yan et al., 2015), directly supporting this functional role. Therefore, we propose a concrete molecular bridge (“mitochondrial/PCD dysfunction → biomarker upregulation → FcγR pathway activation”) that elucidates how this pathway contributes to KD pathology, potentially independent of the baseline genetic architecture of the FCGR locus. This mechanistic insight not only deepens the understanding of KD pathogenesis but also highlights these biomarkers and their associated pathways as potential therapeutic targets.

### 4.5 Limitations

This study has several limitations. First, our bioinformatics analysis was primarily based on transcriptomic data from peripheral blood. Consequently, it lacked direct investigation into the specific expression patterns and functional mechanisms of the identified biomarkers within target tissues, such as the vascular endothelium, which is central to Kawasaki disease pathology. Second, the cohort for clinical validation via RT-qPCR was relatively small. The lack of significant differential expression for biomarkers like NFE2 and CSF3R in our clinical samples suggests that their expression may be influenced by factors including ethnic heterogeneity, disease phase, or sample type, underscoring the need for validation in larger, multi-center cohorts. Third, the therapeutic candidates identified through drug prediction and molecular docking, such as Decitabine and Ibuprofen, were only assessed *in silico*. Their actual efficacy and mechanisms of action in the context of Kawasaki disease require further validation through *in vitro* and *in vivo* functional experiments. Future studies should focus on elucidating the functional roles of these biomarkers in mitochondrial-programmed cell death crosstalk within relevant cell and animal models, alongside expanded clinical validation.

## 5 Conclusion

In conclusion, this study identifies CD177, MMP9, NFE2, CSF3R, and SOCS3 as a panel of biomarkers correlating with both PCD and mitochondrial function in KD. These biomarkers are not merely associated with KD but appear to form a cohesive network that operates within the central pathogenic axis of mitochondrial dysfunction and dysregulated PCD, driving vascular pathology. The diagnostic nomogram based on these markers shows excellent predictive value. Clinical validation confirmed CD177, MMP9, and SOCS3 as promising biomarkers, while NFE2 and CSF3R present intriguing new candidates warranting further investigation. Our findings provide a novel, mechanism-integrated perspective on KD biomarker discovery, offering potential new avenues for diagnosis and targeted therapeutic strategies aimed at the intersection of mitochondrial function and PCD.

## Data Availability

The original contributions presented in the study are publicly available. These data can be found in the NCBI Gene Expression Omnibus (GEO) repository under the accession numbers GSE73461 and GSE68004.
