# Supplementary material for: Identification of programmed cell death and mitochondria correlated biomarkers for Kawasaki disease by integrated bioinformatics and machine-learning algorithms with RT-qPCR verification
Source: Front Genet. 2026 Feb 11;17:1677146. doi: 10.3389/fgene.2026.1677146 (PMC12931890; doi:10.3389/fgene.2026.1677146)
Supplement: Supplementary file 1 [file Table1.docx]

Supplementary Material

# Supplementary Tables

**Table S1. Primer sequence table of this study.**

| **primer** | **sequences** |
| --- | --- |
| CD177 F | TACCCCCACGATTCTTCACC |
| CD177 R | TGGGAGAAAGGACAGGGTCT |
| MMP9 F | GGTGATTGACGACGCCTTTG |
| MMP9 R | GGACCACAACTCGTCATCGT |
| NFE2 F | CTGCCTTTAGCCAGGAAAACAG |
| NFE2 R | TGGGGCTCAAATGATGGCTC |
| CSF3R F | TCTGGACCAACGCTCAGAAC |
| CSF3R R | AACAGGCCCAGGATGATGTG |
| SOCS3 F | GCGAAGGCTCCTTTGTGGA |
| SOCS3 R | GGTCCCGAATCGAAGTCTCC |
| GAPDH F | CGAAGGTGGAGTCAACGGATTT |
| GAPDH R | ATGGGTGGAATCATATTGGAAC |

**Table S2. The intersection result information table of PCD subtype genes and KD differential genes.**

| **Subtype** | **Total_Genes** | **Intersection_Genes** | **Intersection_Count** |
| --- | --- | --- | --- |
| Apoptosis | 580 | ACKR3,ARL6IP5,ARRB2,ATM,BCL3,CASP1,CASP4,CASP5,CD14,CD24,CD27,CD3E,CD44,CD5,CD74,CDKN2D,CEBPB,CFLAR,CTNNA1,CTSC,CYP1B1,DDIAS,DDX47,DYRK2,ERO1A,FHIT,FYN,GNAI3,GPER1,GRINA,HIP1,HMGB2,HSPA1A,HSPA1B,IFI16,IL1B,ITGAM,ITM2C,ITPRIP,JAK2,LCK,LRRK2,LTBR,LY96,MAP3K5,MCL1,MIR21,MMP9,NACC2,NBN,NFE2L2,PEA15,PLAUR,PLEKHF1,PLSCR3,PPP1R13B,PPP1R15A,PPP3CC,PRKCD,PTEN,PTPN1,PTPRC,PTTG1IP,PYCARD,RHOT1,RIPK3,RPL11,RPL26,RPS3,S100A8,S100A9,SHISA5,SIAH2,SOD2,SORT1,SP100,STK3,STRADB,TCF7L2,TLR4,TMEM109,TNF,TNFRSF1A,TNFSF10,TYROBP,VNN1,YWHAH | 87 |
| Ferroptosis | 88 | ACSL1,ACSL4,ALOX12,ATG7,CD44,CYBB,FTH1,G6PD,LPCAT3,NFE2L2,PEBP1,PGD,SAT1,SLC40A1 | 14 |
| Immunogenic.cell.death | 34 | CASP1,CD4,CD8A,CXCR3,ENTPD1,IFNGR1,IL17RA,IL1B,LY96,MYD88,PRF1,TLR4,TNF | 13 |
| Netoticcelldeath | 8 | ELANE,MPO,CAMP,PADI4 | 4 |
| Cuproptosis | 19 | NFE2L2,MTF1 | 2 |
| Oxeiptosis | 5 |  | 0 |
